# The Application of Nitrous Oxide and Air, or Nitrous Oxide and Oxygen, under Pressure, to Produce Anæsthesia in Persons for Dental and Surgical Operations

**Published:** 1883-09

**Authors:** E. P. Howland

**Affiliations:** Washington, D. C.


### Independent Practitioner.

Vol. IV. September, 1883. No. 9.

### Original Communications.

### THE APPLICATION OF NITROUS OXIDE AND AIR, OR NITROUS OXIDE AND OXYGEN, UNDER PRESSURE, TO PRODUCE ANÆSTHESIA IN PERSONS FOR DENTAL AND SURGICAL OPERATIONS.

BY DR. E. P. HOWLAND, WASHINGTON, D. C.

Read before the Biological Section of the American Association for the Advancement of Science, at Minneapolis, Minn., August 21st, 1883

From my own experiments and the practical operations made in Paris, I believe that the administration of nitrous oxide and oxygen in condensed air chambers will yet supersede the use of ether and chloroform in prolonged dental and surgical operations, as nitrous oxide has already superseded them in ordinary dental operations. The reason why nitrous oxide cannot be used for prolonged operations, as ordinarily administered, is, that the blood does not obtain any oxygen from the nitrous oxide, the latter being exhaled from the lungs undecomposed, and if breathed without air or oxygen will produce asphyxia.

I have administered nitrous oxide for dental and surgical operations to over 30,000, and found that when pure nitrous oxide is administered without any admixture of air, the average time of producing anæsthesia is fifty seconds, and the average time from the first commencement of breathing the gas until the return of consciousness is two minutes. I have administered pure nitrous oxide in over 300 surgical operations in the city of Washington, the longest time a patient was unconscious being thirty-five minutes during a capital operation. The operation was performed by Dr. Bliss, and Dr. Stuart.

The skill and experience required in administering pure nitrous oxide for these operations can be acquired by but a few persons, as when the patient has become anæsthetized air has to be breathed to prevent asphyxia, and before consciousness returns again, the interval required for the latter varying from one-fourth to one-half a minute. Pure nitrous oxide will, therefore, never be made practical for prolonged operations. In my experiments on animals, having them breathe pure nitrous oxide, death generally occurs in about 2½ minutes. If air or oxygen is mixed with nitrous oxide in sufficient quantity to prevent asphyxia it will not produce anæsthesia. But it can be mixed with equal quantities of air and breathed from a gas-bag in a condensed air chamber at fifteen pounds pressure per square inch, or mixed with oxygen in the proportion of eighty-five parts of nitrous oxide to fifteen parts of oxygen in a chamber where the surrounding air is compressed to five pounds pressure per square inch, and the mixture can be breathed an indefinite length of time, without danger or injury, producing perfect anæsthesia and perfectly oxygenating the blood. The compressed air in the chamber is merely used to compress the mixture in the gas-bag into a smaller space, so that the patient can breathe sufficient nitrous oxide to produce anæsthesia, and sufficient air or oxygen to oxygenate the blood.

The application of nitrous oxide and air or oxygen as an anæsthetic agent for prolonged operations, has but a very recent history.

On the 2nd day of February, 1878, Paul Bert announced to the Biological Society of Paris his discovery, as the result of theoretical deductions, of a process by which the anæsthetic properties of nitrous oxide could be used without danger of asphyxia. No experience had yet been obtained. When Paul Bert passed from theory to practice and experimented upon animals, he saw all his experiments crowned with the greatest success. The results that he had announced in advance were realized beyond his hopes. The animals were plunged into a profound insensibility which could be indefinitely prolonged, and during which none of their vital functions were disturbed. The method was excellent in every respect. Encouraged by this result, Paul Bert resolved to apply to man the same method, and to perform, by means of it, the longest and most painful operations. In a note, under date of Nov. 11, 1878, he appeals to surgeons ; he says : "I am now authorized by my experience with animals to commend urgently to surgeons the use of nitrous oxide and oxygen, under pressure, with the view of producing insensibility of long duration.

"I can assure them that by measuring, as I have indicated, the barometric pressure and the centesimal composition of the mixture so as to have for the nitrous oxide the tension of the atmosphere, and for the oxygen at least the normal tension of the surrounding area, they will obtain an insensibility and a muscular relaxation as complete as they may desire, followed by an immediate return to sensibility and perfect comfort.

"There is even a singular ease in applying this agent, for should there be slight inequalities in its effect upon different individuals, as must necessarily be the case on account of special susceptibilities, the barometric pressure need only be slightly increased or diminished, as is very easily done by means of regulating cocks.

"I know there are some difficulties to be overcome in the construction of the chambers for the application of this new anæsthetic ; but for me, as a physiologist, it ought to be enough to have pointed out the agent, so marvelous and so easily applicable, shown the immense advantage of its use, and convinced, among other things, of its harmlessness."

Two Parisian hospital surgeons—Dr. Leon Labbe and Dr. Pean—responded to this appeal. The first operation, performed Feb. 15, 1879, by Dr. L. Labbe, was all-sufficient to demonstrate the excellence of the method, and the precious advantages to be derived from it.

Subsequent operations, performed partly by Dr. Labbe and partly by Dr. Pean, have given more eclat to that demonstration. These expectations of Paul Bert were then completely realized, nitrous oxide and oxygen had definitely entered the domain of grand surgery, and the superiority of this anæsthetic agent over ether and chloroform had been forever demonstrated. His success was complete, and in a new note, addressed to the Academy of Sciences of Paris, Paul Bert mentioned the happy results of the first operations performed by the able surgeons just named. The surgeons, who had, at first, doubtless been frightened by the necessity of staying in compressed air, or by the sight of the metallic chamber in which they should perform their operations, were then interested by this method, to which they had previously manifested a strange indifference.

Dr. Perier, surgeon of the hospital of St. Antoine, began on May 7, 1880, to operate with nitrous oxide and oxygen, and on May 20th, Dr. Ledentu, surgeon of the hospital of St. Louis, made his first operation with Paul Bert's anæsthetic process. All these operations succeeded perfectly, as well as many performed by other French surgeons. In Paris several hundred operations have now been performed, and all have been crowned with the most brilliant success. The method is in a good way, and will no doubt in a short time supplant ether and chloroform, at least in hospital and city practice, and I think that the greater facilities in administering this agent, regulating the pressure and purifying the air from infectious germs in the air chamber that I have devised, will greatly facilitate its introduction.

The general conclusions to which we are led by the foregoing exposition may be formulated as follows:

(1.) Nitrous oxide, administered under pressure, and mixed with oxygen, produces within a few seconds a profound insensibility.

(2.) Under these conditions, life may be indefinitely sustained without the least danger of asphyxia.

(3.) In augmenting or diminishing the pressure, the degree of anæsthesia may be regulated at will, and with mathematical precision. Therefore, there is no danger of any of the accidents incurred through the use of ether or chloroform.

(4.) When inhalation of nitrous oxide and oxygen is stopped, the patient recovers consciousness in a few seconds, and feels no consequent discomfort.

(5.) Nitrous oxide is merely dissolved in the plasma of the blood, and escapes when inhalation ceases.

(6.) Its use causes no danger to nutrition, and no change in the chemical composition of the organs, or cessation of their functions.

(7.) The action of compressed air upon the operator and his assistants need not be feared.

Compressed air is very efficacious in the treatment of catarrh of the mucous membrane of the nose, the Eustachian tube and the respiratory organs generally.

(8.) By reason of these facts, nitrous oxide and oxygen is proven to be superior to ether or chloroform, whether we consider its profound anæsthetic effect or its freedom from injurious results.

(9.) If the pressure of the air chamber is rightly and properly regulated it is absolutely impossible for the patient to run any risk by anæsthesia alone.

(10) In all that concerns the application of nitrous oxide and oxygen to surgery, the scientific phase may be said to be exhausted, and this anæsthetic agent should be henceforth used for operations of infinite duration, instead of ether and chloroform.

Dr. Howland at the close of his paper, illustrated his points by the aid of suitable apparatus in the following manner:

A small box was provided to serve as an air chamber in which glass windows and a door were set and properly sealed. In this box was placed a live chicken. Into this chamber, by means of stopcocks and rubber tubes, the air being previously exhausted, pure nitrous oxide was first forced from a gas-bag, producing insensibility in the chicken in the space of forty seconds. This was immediately followed by the mixture of nitrous oxide and oxygen, introduced from an iron reservoir, by means of which anæsthesia was sustained for seven minutes, as an illustration of its power, at a pressure of four pounds to the square inch. The chicken was then removed from the chamber, feathers were plucked from it for five seconds subsequent to its removal without eliciting signs of pain, and within twelve seconds consciousness was fully restored.

It was again placed in the box and pure nitrous oxide was introduced, with the result of causing death in fifty seconds. On removal and decapitation of the bird, its blood was found to be in a non-arterialized condition.

